# Assessment of Firearm Storage Practices in the US, 2022

**DOI:** 10.1001/jamanetworkopen.2023.1447

**Published:** 2023-03-02

**Authors:** Michael D. Anestis, Jayna Moceri-Brooks, Rachel L. Johnson, Craig J. Bryan, Ian H. Stanley, Jessica T. Buck-Atkinson, Justin C. Baker, Marian E. Betz

**Affiliations:** 1 New Jersey Gun Violence Research Center, Piscataway; 2 School of Public Health, Rutgers, The State University of New Jersey, Piscataway; 3 Department of Biostatistics and Informatics, Colorado School of Public Health, University of Colorado Anschutz Medical Campus, Aurora; 4 Department of Psychiatry and Behavioral Health, The Ohio State University College of Medicine, Columbus; 5 Department of Emergency Medicine, School of Medicine, University of Colorado Anschutz Medical Campus, Aurora; 6 Center for COMBAT Research, Department of Emergency Medicine, University of Colorado School of Medicine, Aurora; 7 Firearm Injury Prevention Initiative, University of Colorado Anschutz Medical Campus, Aurora; 8 VA Eastern Colorado Geriatric Research Education and Clinical Center, Denver

## Abstract

**Importance:**

Secure firearm storage may help reduce firearm injury and death. Broad implementation requires more granular assessments of firearm storage practices and greater clarity on circumstances that may prevent or promote the use of locking devices.

**Objective:**

To develop a more thorough understanding of firearm storage practices, obstacles to using locking devices, and circumstances in which firearm owners would consider locking unsecured firearms.

**Design, Setting, and Participants:**

A cross-sectional, nationally representative survey of adults residing in 5 US states who owned firearms was administered online between July 28 and August 8, 2022. Participants were recruited via probability-based sampling.

**Main Outcomes and Measures:**

Firearm storage practices were assessed via a matrix provided to participants in which firearm-locking devices were described both via text and images. Locking mechanisms (key/personal identification number [PIN]/dial vs biometric) were specified for each type of device. Obstacles to the use of locking devices and circumstances in which firearm owners would consider locking unsecured firearms were assessed via self-report items developed by the study team.

**Results:**

The final weighted sample included 2152 adult (aged ≥18 years), English-speaking firearm owners residing in the US; the sample was predominantly male (66.7%). Among the 2152 firearm owners, 58.3% (95% CI, 55.9%-60.6%) reported storing at least 1 firearm unlocked and hidden, with 17.9% (95% CI, 16.2%-19.8%) reporting storing at least 1 firearm unlocked and unhidden. Gun safes were the most frequently used device both among participants who use keyed/PIN/dial locking mechanisms (32.4%; 95% CI, 30.2%-34.7%) and those who use biometric locking mechanisms (15.6%; 95% CI, 13.9%-17.5%). Those who do not store firearms locked most frequently noted a belief that locks are unnecessary (49.3%; 95% CI, 45.5%-53.1%) and a fear that locks would prevent quick access in an emergency (44.8%; 95% CI, 41.1%-48.7%) as obstacles to lock usage. Preventing access by children was the most often reported circumstance in which firearm owners would consider locking unsecured firearms (48.5%; 95% CI, 45.6%-51.4%).

**Conclusions and Relevance:**

In this survey study of 2152 firearm owners, consistent with prior research, unsecure firearm storage was common. Firearm owners appeared to prefer gun safes relative to cable locks and trigger locks, indicating that locking device distribution programs may not match firearm owners’ preferences. Broad implementation of secure firearm storage may require addressing disproportionate fears of home intruders and increasing awareness of the risks associated with household firearm access. Furthermore, implementation efforts may hinge on broader awareness of the risks of ready firearm access beyond unauthorized access by children.

## Key Points

**Question:**

What types of locking devices are most frequently used on firearms by firearm owners in the US?

**Findings:**

In this survey study with a weighted sample of 2152 adults, the results indicated gun safes were the most frequently used device by the respondents. Believing locks are unnecessary and concerns about access speed were the most frequent obstacles; concern about child access was the most common reason for considering locking unlocked firearms.

**Meaning:**

The findings of this survey suggest that increasing the use of secure storage may require increasing access to safes, ameliorating fears about speed of access, and clarifying the risks associated with unlocked firearms.

## Teaser

This survey study examines firearm storage practices and factors that may prevent or promote the use of locking devices among a nationally representative sample of firearm-owning US adults.

## Introduction

Firearm injury and death are substantial public health concerns in the US. Research has noted that access to firearms is associated with increased likelihood of injury and death.^1,2,3^ With an estimated 393 million privately owned firearms in circulation^4^ and a constitutionally defined right to firearm access, reducing access nationally is difficult.^5^ Therefore, some scientific and clinical efforts have focused on mitigating risk associated with firearm access in homes.^6^

Unsecure home firearm storage is associated with further increased risk of firearm death,^7,8,9^ and the promotion of secure firearm storage (eg, with a locking device) may help reduce firearm injury and death. Despite its potential value, secure firearm storage is uncommon. In a nationally representative sample of firearm owners (n = 2072), 65.3% reported storing at least 1 firearm unlocked.^10^ Similar patterns have emerged in studies examining families with and without children in the home, older adults, and military veterans.^11,12,13,14^ One study^15^ reported that 40% of firearm owners noted at least occasionally storing firearms in their vehicles, with more than 15% of those individuals storing firearms loaded and unlocked. Furthermore, some high-risk communities (eg, firearm-owning military service members with a history of suicidal ideation) are particularly prone to unsecure firearm storage.^16,17^ Together, these findings highlight that large proportions of firearm owners store their firearms unsecured.

Prior research on firearm storage has been limited in the degree of detail assessed regarding the types of storage devices and in reliance of studies on text descriptions of storage devices that assume respondents interpret labels accurately. For instance, many studies combine aspects of storage practices (eg, loaded and locked status^11,13^) or types of devices (eg, gun safes, lock boxes^17^) into a single item, precluding detailed understanding of specific storage tendencies. A range of storage devices exists and, as such, merely ascertaining whether a firearm is locked says little about the specific locking devices used. This shortcoming obscures our understanding of firearm storage practices nationwide and limits our understanding of storage preferences among specific firearm owner groups. Furthermore, to our knowledge, no studies have considered specific locking devices (eg, safe, trigger lock) separate from locking mechanisms (eg, key/personal identification number [PIN]/dial vs biometric), leaving knowledge gaps about the distribution of locking mechanisms across firearm-owning households. Developing a more granular understanding of firearm storage practices and motives can assist in developing locking device distribution and secure firearm storage messaging efforts that reflect the actual needs and perceptions of firearm-owning communities, thereby potentially increasing their effectiveness.

Prior studies examining firearm storage practices have generally lacked assessment of factors that might be associated with the use of different storage practices.^10,11,12,13,14^ Understanding how individuals store their firearms, the obstacles that prevent them from adopting secure storage, and what circumstances might prompt the use of locking devices could inform policy and practice regarding distributing locking devices. Individuals may, for example, prefer different locking mechanisms depending on the type of firearms they own and their reasons for owning those firearms. They also may resist specific forms of locking devices due to concerns about whether the device will impede their intended use of the firearm (eg, quick access in case of home invasion) or consider adopting locked storage practices under specific sets of circumstances.

In this study, we aimed to expand on prior research by providing a nuanced description of firearm storage practices within a nationally representative sample of firearm owners. In contrast to prior studies that used verbal descriptions of locking devices, we used a combination of image and text descriptions of locking devices to assess firearm storage practices. We further allowed respondents to express their preferences about locking mechanisms separate from the locking device itself. In addition, we aimed to characterize obstacles to secure firearm storage and circumstances that may prompt adoption of various storage practices. Our findings could help provide greater clarity on the landscape of firearm storage practices while also informing policies and programs that involve the distribution of firearm-locking devices.

## Methods

We conducted an online survey between July 28 and August 8, 2022, recruiting firearm-owning participants from Ipsos KnowledgePanel (KP), a probability-based panel developed to be representative of English-speaking US adults (aged ≥18 years). The survey included an initial recruitment effort (3908 fielded, 2105 completed, completion rate: 53.9%; qualification rate: 97.5%) as well as an augment of military veterans (173 fielded, 102 completed, completion rate: 59.0%; 98.0% qualification rate). Qualification rate represents the percentage of individuals contacted about participation who met inclusion criteria for the protocol (aged ≥18 years, residing within the US). All participants provided informed consent, and participants were compensated with points that count toward an Ipsos incentive program. The protocol was reviewed and approved by the University of Colorado Institutional Review Board as well as the Department of Defense Human Research Protection Office. The study followed the American Association for Public Opinion Research (AAPOR) reporting guideline.

Ipsos provided study-specific poststratification weights through their patented method that was developed to create samples behaving as expected by principles of the equal probability selection method. Data on active members of the KP pool were weighted with regard to geodemographic benchmarks for the US Census Bureau American Community Survey, the latest Census Bureau Current Population Survey, and participant responses. Design weights for KP firearm owners were ranked to control for demographic characteristics (gender, age, race and ethnicity, census region, metropolitan status, educational level, household income, and gender by age [ie, grouped simultaneously]) by veteran status. Race and ethnicity were included in weighting procedures to ensure that the demographic profile of the final dataset was representative of US firearm owners.

Types of firearms owned was assessed through a series of questions. The first asked, “How many handguns are in/near your home?” The second asked, “How many long guns are in/near your home?” To assess reasons for firearm ownership, participants were presented with a matrix listing 5 potential reasons for ownership: home protection, carry/protection out of home, hunting/sport, occupation (eg, law enforcement, security), and as heirloom/collectible. Participants could then indicate whether they owned any handguns or long guns for any of these reasons or could indicate that they do not own any firearms for those purposes.

To assess current firearm storage practices, participants were presented with a series of items depending on which types of firearms they reported owning for specific reasons. For each selected reason for firearm ownership, participants were presented with the following wording: “What storage/staging device(s) do you currently use for that/those firearm(s) used for [specific purpose]?” If the participant did not report owning any firearms for any of the reasons listed in the previous question, they were instead presented with the following text: “You indicated that you do not have any firearms in/near your home for any of the purposes listed in the previous question, but you did indicate that you have firearms in/near your home. What storage/staging device(s) do you use for that/those firearm(s)?” Participants were then presented a matrix listing a variety of storage devices, broken down by locking mechanism (key, PIN, dial vs biometric), with text descriptions of each method paired with an image representing that method (eFigure in Supplement 1). Participants were also presented with the option of selecting “unlocked, hidden” and “unlocked, not hidden.”

To assess reasons for current storage practices, participants who reported any of the secure firearm storage options from the previous item were asked “What are the reasons you currently use storage/staging locking devices?” Answer choices included “prevent theft,” “prevent unauthorized access by an adult household member,” “prevent access by an adolescent/teenager,” “prevent access by a child (younger than adolescent/teenager),” “keep firearm in good condition,” and “other.” Individuals who reported storing any firearms unlocked were asked, “Are there any circumstances where you would consider using a locking device for the firearm(s) you indicated are currently unlocked?” Answer choices for this item mirrored those for the previous item.

To assess obstacles to using locking devices, participants were asked, “Do you use locking devices on all of your firearms?” Those who answered no were asked, “Why not?” The participant then selected from the following answer choices: “too expensive,” “not sure which one to buy,” “no store near me to buy one,” “takes too long to access firearm in an emergency,” “too easy to break into,” “will damage my firearm(s),” “don’t need one,” or “other.”

### Statistical Analysis

Analyses were conducted in R, version 4.2.1 (R Foundation for Statistical Computing) with the R package survey.^18^ A weight variable was provided by Ipsos, with study-specific poststratification weights used to account for any differential nonresponse that may have occurred. Summary statistics are presented with weighted means and weighted SDs for continuous variables and with weighted percentages and 95% CIs for categorical variables. Weighted percentages or means representing fewer than 30 participants are not presented because this estimate is not stable due to small sample size.

## Results

The final weighted sample included 2152 adult (aged ≥18 years), English-speaking firearm owners residing in the US. The sample was predominantly male (66.7% vs 33.3% female; 95% CI, 64.3%-69.0%) and non-Hispanic White (75.6%; 95% CI, 73.3%-77.8%). Most had attended at least some college (63.9%; 95% CI, 61.5%-66.3%) and nearly half (44.3%; 95% CI, 42.0%-46.7%) reported an annual household income of $100 000 or higher. Full demographic information is available in Table 1.

**Table 1.** Sample Demographic Characteristics

Sociodemographic variable	Weighted % (95% CI)^a^
Age, weighted mean (SD), y	51.9 (16.4)
Educational level	
No high school diploma or GED	6.0 (4.7-7.8)
High school graduate	30.0 (27.8-32.4)
Some college or associate degree	31.8 (29.7-34.1)
Bachelor degree	18.9 (17.3-20.7)
Graduate degree	13.1 (11.8-14.7)
Race and ethnicity^b^	
Hispanic	10.3 (8.7-12.1)
Non-Hispanic	
Black	9.4 (8.1-10.9)
White	75.6 (73.3-77.8)
Multiple	1.8 (1.4-2.3)
Other	3.0 (2.1-4.2)
Gender	
Male	66.7 (64.3-69.0)
Female	33.3 (31.0-35.7)
Annual household income, $	
<10 000	NR
10 000-24 999	6.7 (5.6-8.0)
25 000-49 999	15.2 (13.5-17.1)
50 000-74 999	17.4 (15.6-19.3)
75 000-99 999	14.8 (13.2-16.6)
100 000-149 999	20.0 (18.2-22.0)
≥150 000	24.3 (22.3-26.4)
Marital status	
Married	66.2 (63.8-68.5)
Widowed	4.5 (3.7-5.5)
Divorced	10.2 (8.9-11.6)
Separated	NR
Never married	18.0 (16.0-20.2)
MSA category	
Nonmetropolitan	21.1 (19.1-23.1)
Metropolitan	78.9 (76.9-80.9)

Abbreviations: MSA, metropolitan statistical area; NR, not reported.

^a^ Weighted percentage estimate and 95% CI representing fewer than 30 participants are unstable and therefore not reported.

^b^ Race and ethnicity data were derived from Ipsos KnowledgePanel user profile information and rely on self-report by participants. Categories are grouped in the survey as shown here.

Regarding firearm ownership, most respondents (64.8%; 95% CI, 62.5%-67.1%) reported owning both handguns and long guns, with 25.0% (95% CI, 23.0%-27.2%) reporting only owning handguns and 10.2% (95% CI, 8.9%-11.6%) reporting only owning long guns (Table 2). Nearly all handgun owners (92.6%; 95% CI, 91.2%-93.7%) reported owning handguns for home protection and most (59.5%; 95% CI, 57.0%-62.0%) reported owning for carrying/protection out of home. Among long gun owners, hunting (73.2%; 95% CI, 70.7%-75.5%) was the most commonly reported reason for ownership, followed by home protection (57.1%; 95% CI, 54.3%-59.7%) and owning as an heirloom or collectible (44.5%; 95% CI, 41.8%-47.2%) (Table 2).

**Table 2.** Firearm Ownership Characteristics

Firearm variable	Weighted % (95% CI)^a^
No. of handguns, weighted mean (SD)	3.4 (2.9-3.8)
If handgun owner, No. of handguns, weighted mean (SD)	3.7 (3.3-4.2)
No. of long guns, weighted mean (SD)	3.4 (3.0-3.8)
If long gun owner, No. of long guns, weighted mean (SD)	4.5 (4.0-5.0)
Firearm ownership	
Owns only handgun(s)	25.0 (23.0-27.2)
Owns only long gun(s)	10.2 (8.9-11.6)
Owns both handgun(s) and long gun(s)	64.8 (62.5-67.1)
Purpose of handgun(s) (select all that apply)	
Home protection	92.6 (91.2-93.7)
Carry/protection out of home	59.5 (57.0-62.0)
Hunting	20.0 (18.1-22.0)
Occupation	6.6 (5.4-8.0)
Heirloom/collectible	24.9 (22.8-27.1)
Purpose of long gun(s) (select all that apply)	
Home protection	57.1 (54.3-59.7)
Carry/protection out of home	7.8 (6.3-9.5)
Hunting	73.2 (70.7-75.5)
Occupation	3.0 (2.2-4.1)
Heirloom/collectible	44.5 (41.8-47.2)
If currently using storage/locking device(s) for at least 1 firearm, reason(s) for using (select all that apply)^b^	
Prevent theft	66.0 (63.2-68.7)
Prevent unauthorized access by an adult household member	32.2 (29.5-34.9)
Prevent access by an adolescent/teenager	42.0 (39.1-44.8)
Prevent access by a child	53.3 (50.4-56.1)
Keep firearm in good condition	55.7 (52.8-58.5)
Other	4.7 (3.7-6.0)
If at least 1 firearm is currently stored without storage/locking device(s), are there any circumstances where you would consider using a locking device for the firearm(s) that are currently unlocked? (select all that apply)^c^	
Prevent theft	36.9 (34.1-39.8)
Prevent unauthorized access by an adult household member	21.3 (18.9-23.9)
Prevent access by an adolescent/teenager	36.7 (33.9-39.6)
Prevent access by a child	48.5 (45.6-51.4)
Keep firearm in good condition	15.6 (13.6-17.9)
Other	3.1 (2.2-4.2)
Uses locking device on all firearms	43.9 (41.1-46.8)
If not, why not? (select all that apply)	
Too expensive	NR
Not sure which one to buy	5.4 (3.8-7.5)
No store near me to buy one	NR
Takes too long to access firearm in an emergency	44.8 (41.1-48.7)
Too easy to break into	NR
Will damage my firearm(s)	NR
Don't need one	49.3 (45.5-53.1)
Other	15.4 (12.9-18.4)

^a^ Weighted percentage estimate and 95% CI representing fewer than 30 participants are unstable and therefore not reported.

^b^ This variable only applies to those who have at least 1 firearm stored with a storage/locking device (1473 of 2152).

^c^ This variable only applies to those who have at least 1 firearm stored without a storage/locking device (1440 of 2152); note that there is overlap between this group and the 1473 individuals using a storage/locking device because participants could respond separately for each firearm and the different firearm storage methods they use.

More than half of the sample (58.3%; 95% CI, 55.9%-60.6%) (Table 3) reported storing at least 1 firearm unlocked and hidden, with 17.9% (95% CI, 16.2%-19.8%) reported storing at least 1 firearm unlocked and unhidden. There was limited variability across reasons for ownership or type of firearms owned with respect to storing firearms unlocked and hidden. Individuals who only owned handguns reported storing firearms unlocked and unhidden less frequently vs individuals who owned both handguns and long guns (9.9%; 95% CI, 7.3%-13.3% vs 22.1%; 95% CI, 19.7%-24.6%).

**Table 3.** Firearm Storage Practices Broken Down by Full Sample, Reasons for Ownership, and Types of Firearms Owned

Firearm storage	Weighted % (95% CI)^a^								
	Overall (N = 2152)	Reason for firearm ownership					Type of firearm owned		
		Home protection (n = 1927)	Carry/out of home (n = 1109)	Hunting (n = 1279)	Occupation (n = 141)	Heirloom (n = 937)	Handgun only (n = 515)	Long gun only (n = 242)	Both types (n = 1394)
Unlocked, hidden	58.3 (55.9-60.6)	59.4 (56.9-61.9)	64.4 (61.1-67.5)	57.4 (54.3-60.5)	62.4 (52.9-71.0)	62.4 (58.9-65.8)	56.3 (51.3-61.1)	55.2 (48.1-62.1)	59.5 (56.5-62.4)
Unlocked, not hidden	17.9 (16.2-19.8)	18.9 (17.0-21.0)	24.1 (21.3-27.0)	21.7 (19.3-24.4)	23.7 (16.6-32.8)	22.7 (19.8-25.8)	9.9 (7.3-13.3)	NR	22.1 (19.7-24.6)
Keyed/PIN/dial									
Cable lock	19.3 (17.5-21.4)	19.9 (18.0-22.1)	22.2 (19.5-25.1)	20.5 (18.1-23.1)	24.1 (17.0-33.1)	18.4 (15.8-21.4)	19.8 (16.1-24.2)	NR	20.8 (18.4-23.3)
In-vehicle lock	7.1 (5.9-8.4)	7.8 (6.5-9.3)	12.6 (10.6-15.0)	7.9 (6.4-9.7)	NR	8.9 (7.0-11.2)	NR	NR	9.5 (7.8-11.4)
Trigger lock	11.8 (10.4-13.4)	12.1 (10.6-13.9)	12.8 (10.7-15.1)	13.4 (11.5-15.7)	NR	14.0 (11.7-16.7)	8.6 (6.3-11.7)	NR	13.9 (11.9-16.0)
Gun safe	32.4 (30.2-34.7)	33.3 (31.0-35.7)	40.0 (36.8-43.3)	44.6 (41.6-47.7)	45.8 (36.6-55.3)	43.6 (40.1-47.2)	7.0 (4.8-10.1)	16.5 (11.4-23.2)	44.7 (41.7-47.7)
Clamshell	5.9 (4.8-7.3)	6.4 (5.2-7.9)	9.1 (7.2-11.4)	5.7 (4.3-7.4)	NR	6.9 (5.1-9.3)	NR	NR	6.6 (5.2-8.4)
Gun cabinet	11.4 (9.9-13.1)	11.7 (10.1-13.5)	13.1 (10.9-15.6)	16.9 (14.6-19.4)	NR	16.8 (14.2-19.8)	NR	NR	15.1 (13.0-17.5)
Small lockbox/hard case	13.9 (12.4-15.6)	14.5 (12.8-16.3)	17.8 (15.4-20.4)	13.2 (11.3-15.3)	NR	13.0 (10.8-15.6)	15.8 (12.5-19.7)	NR	15.2 (13.2-17.4)
Other key/PIN/dial locking device	2.5 (1.9-3.4)	2.5 (1.8-3.3)	2.9 (2.0-4.2)	3.4 (2.5-4.6)	NR	3.5 (2.4-5.1)	NR	NR	3.2 (2.3-4.3)
Biometric									
Cable lock	1.7 (1.2-2.5)	1.9 (1.3-2.7)	NR	NR	NR	NR	NR	NR	2.4 (1.6-3.6)
In-vehicle lock	3.5 (2.7-4.7)	3.8 (2.9-5.0)	6.3 (4.8-8.3)	3.8 (2.7-5.3)	NR	3.9 (2.5-5.9)	NR	NR	4.8 (3.5-6.4)
Trigger lock	3.8 (3.0-4.8)	4.0 (3.1-5.1)	5.0 (3.8-6.7)	4.4 (3.3-5.8)	NR	4.3 (3.0-6.1)	NR	NR	4.1 (3.1-5.5)
Gun safe	15.6 (13.9-17.5)	16.8 (15.0-18.9)	20.5 (17.9-23.3)	16.5 (14.2-19.1)	29.7 (22.0-38.6)	15.7 (13.3-18.5)	15.6 (12.4-19.5)	NR	17.9 (15.7-20.4)
Clamshell	NR	NR	NR	NR	NR	NR	NR	NR	NR
Gun cabinet	2.8 (2.1-3.8)	3.0 (2.2-4.0)	3.3 (2.3-4.8)	3.6 (2.6-5.0)	NR	NR	NR	NR	4.1 (3.1-5.5)
Small lockbox/hard case	8.3 (7.0-9.7)	8.7 (7.4-10.3)	11.2 (9.2-13.5)	8.2 (6.7-10.1)	NR	8.4 (6.7-10.7)	8.8 (6.3-12.2)	NR	9.2 (7.6-11.1)
Other key/PIN/dial locking device	1.7 (1.2-2.5)	1.9 (1.3-2.7)	NR	NR	NR	NR	NR	NR	1.8 (1.1-2.7)

Abbreviations: NR, not reported; PIN, personal identification number.

^a^ Weighted percentage estimate and 95% CI representing fewer than 30 participants are unstable and therefore not reported. In addition, 9 participants reported owning a firearm for another reason; since this entire column was not reported due to all percentages representing cell sizes of less than 30, this column is not shown.

Gun safes were the most frequently reported storage approach, both among devices that use keyed/PIN/dial locking mechanisms (32.4%; 95% CI, 30.2%-34.7%) and those that use biometric locking mechanisms (15.6%; 95% CI, 13.9%-17.5%) (Table 3). Frequency varied meaningfully by type of firearm owned. Individuals who only owned handguns were less likely to use safes vs those who owned both handguns and long guns (7.0%; 95% CI, 4.8%-10.1% vs 44.7%; 95% CI, 41.7%-47.7% for keyed/PIN/dial locks). There was limited variability in the frequency of reports of other storage devices across reasons for firearm ownership and types of firearms owned. Only 19.3% (95% CI, 17.5%-21.4%) of the sample reported using keyed/PIN/dial lock cable locks and 1.7% (95% CI, 1.2%-2.5%) reported using biometric cable locks. Here again, there was minimal variability across reason for ownership and type of firearm owned (Table 3).

The most frequently reported reason for current storage practices were theft prevention (66.0%; 95% CI, 63.2%-68.7%) (Table 2), keeping the firearm in good condition (55.7%, 95% CI, 52.8%-58.5%), and preventing access by a child (aged 0-12 years; 53.3%; 95% CI, 50.4%-56.1%). Preventing a child from accessing firearms was the most frequently reported circumstance in which firearm owners would consider locking currently unlocked firearms (48.5%; 95% CI, 45.6%-51.4%), with 36.9% (95% CI, 34.1%-39.8%) noting that theft prevention and 36.7% (95% CI, 33.9%-39.6%) noting prevention of access by an adolescent/teenager may prompt them to lock their firearms. Among those who do not currently lock all their firearms (56.1% of respondents), the most frequently reported obstacles were beliefs that locks are not needed (49.3%; 95% CI, 45.5%-53.1%) and concerns that locked firearms take too long to access during an emergency (44.8%; 95% CI, 41.1%-48.7%).

## Discussion

In this nationally representative sample, firearm owners reported a variety of firearm storage practices, reasons for specific storage approaches, and obstacles to adopting secure storage. Our findings build on existing research in several ways. First, our assessment tool examined specific types of locking devices instead of using an aggregate locking device variable. Second, we assessed locking mechanism (key/PIN/dial vs biometric) and locking device separately, thereby obtaining greater nuance with respect to respondent preferences. Third, we provided participants with visual images of each storage device to reduce possible confusion surrounding terminology. Collectively, these design features provide an incrementally valuable description of firearm storage practices in a large, nationally representative sample of firearm owners, with follow-up items providing insight into existing obstacles preventing the use of locking devices and circumstances in which firearm owners would consider locking up firearms currently stored unlocked.

Consistent with prior research,^8,9,10,11,12,13^ our results indicated secure firearm storage is uncommon. Approximately two-thirds of the sample reported storing at least 1 firearm unlocked, and that figure varied minimally based on reasons for firearm ownership and types of firearms owned. Among those who reported locking their firearms, owners noted using storage devices that use keyed/PIN/dial locking mechanisms more frequently than devices that use biometric locking mechanisms. Future research should explore the reasons for this trend. Biometric locking devices offer a theoretically more palatable path toward secure firearm storage for individuals who own firearms for protection, as they allow for quicker access without the need to input a combination or find a key. For this method to become more commonly used, however, firearm owners will need to trust that the technology will work reliably when they need to access the firearm.^19^ Nevertheless, their limited use in this sample indicates that uptake of this approach has been blunted and that the more traditional locking mechanisms represent the bulk of the market for firearm-locking devices. From the suicide prevention perspective, biometric devices are less appealing due to the potential speed of access; however, these tools may help prevent child and adolescent suicide by preventing access to family members’ firearms and, furthermore, a biometrically locked device may offer more protection than an unlocked device. As such, there may be clinical value in promoting biometric locks and facilitating access among individuals who own firearms for self-defense. Any effort to promote use of biometric locks—or any other form of locking device—should also be paired with efforts to promote shifts in risk perceptions among firearm owners such that their views better reflect the minimal risk of home intruders, particularly when compared with the risk represented even by securely stored firearms in the home.

Within both categories of locking mechanisms, gun safes were the most frequently used storage approach. The distribution of responses by type of firearm appears to support the notion that safes are more commonly used for long guns, perhaps due to less perceived need for quick access relative to handguns, which are more often owned for protection at home.^16^ Indeed, concerns about ease of access in an emergency were a commonly reported reason for not locking firearms. These data highlight the importance of adjusting risk perceptions among handgun owners and specifically targeting such firearm owners in secure firearm storage promotion efforts. Ultimately, if we cannot prompt population-level changes in risk perceptions such that defensive firearm owners—particularly handgun owners—view violent home invasion as less likely than other violent outcomes stemming from their household firearm access, we must promote secure firearm storage tools that allow quick enough access to assuage fears and facilitate adoption of storage practices that are at least safer than current practices.

Although cable or trigger locks are included in most legal firearm purchases and are distributed widely by a variety of groups (eg, Veterans Affairs Hospitals), only a small proportion of the sample reported using them. This highlights that simply providing locking devices does not ensure use. Furthermore, this emphasizes that building distribution strategies around approaches that do not heavily weight storage preferences will likely result in limited increases in secure firearm storage.^20^

Individuals frequently reported preventing access by children as a reason for current storage approaches and as a motivation for considering locking currently unlocked firearms. This may indicate firearm owners perceive risks associated with child access as outweighing the risk of potential home invasion. Secure firearm storage messaging that helps clarify the risk of unsecured firearms beyond situations involving child access may thus serve as a method for increasing secure storage^17^; however, such messaging must rely on credible sources who deliver their messages via trusted channels.^21,22,23^

### Limitations

The study has limitations. Due to Department of Defense restrictions in place at the time of data collection, active duty service members were excluded from the sample. Although service members represent only a small percentage of the US population and military veterans may serve as a reasonable proxy, their exclusion from the sample nonetheless impacts generalizability of the findings. Our completion rate (ie, 53.9%-59.0%) was acceptable, but not optimal and, therefore, sampling bias and representativeness remain possible concerns. Although our use of visuals likely increased comprehension, we relied on self-report for information. Our sample was also limited in its representation of firearm ownership whose racial identity was not White. Although our granular assessment of firearm-locking practices represents an advance on prior assessments, we restricted our measurement to within-home storage options, thereby precluding any understanding of our outside-of-home options. Finally, our assessment focused entirely on lock status, without any assessment of load status. The primary aim of this project was to better understand locking practices and obstacles to using such devices so as to help better organize efforts to increase the update of this specific component of secure firearm storage. It may be beneficial for future work to incorporate load status into their assessment to help provide more detail that might prove useful in preventing firearm injury and death.

## Conclusions

Our findings provide a detailed description of firearm storage practices among US adults. Furthermore, our results highlight obstacles to secure storage use and circumstances in which firearm owners would consider locking firearms, thereby providing a framework for future research aiming to increase uptake of specific storage approaches.

## Notes

Supplement 1. **eFigure.** Assessment Tool for Firearm-Locking Devices

Supplement 2.
Data Sharing Statement
