# Supplementary material for: Assessment of Firearm Storage Practices in the US, 2022
Source: JAMA Netw Open. 2023 Mar 2;6(3):e231447. doi: 10.1001/jamanetworkopen.2023.1447 (PMC9982690; doi:10.1001/jamanetworkopen.2023.1447)
Supplement: Supplement 1. — eFigure. Assessment Tool for Firearm-Locking Devices [file jamanetwopen-e231447-s001.pdf]

Supplementary Online Content

Anestis MD, Moceri-Brooks J, Johnson RL, et al. Assessment of firearm storage practices in the US, 2022. *JAMA Netw Open*. 2023;6(3):e231447. doi:10.1001/jamanetworkopen.2023.1447

eFigure. Assessment Tool for Firearm-Locking Devices

This supplementary material has been provided by the authors to give readers additional information about their work.

© 2023 Anestis MD et al. *JAMA Network Open.*

eFigure. Assessment Tool for Firearm-Locking Devices

[If handgun OR long gun for any row in Q3] What storage/staging device(s) do you currently use for that/those firearm(s) used for *[purpose]*? *Choose all that apply. [refer to storage chart]*

Note images are only as example and may not represent all devices in that category			
**Storage Type**		**Type**	**Example image**
**Unlocked**			
a. Unlocked, hidden		b. Unlocked, not hidden	
**Locking device with a key, number PIN, or dial**			
c. Cable lock	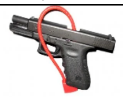	d. In-vehicle lock	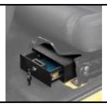
e. Trigger lock	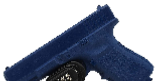	f. Gun safe	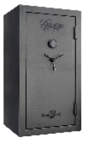
g. Clamshell/" life jacket"	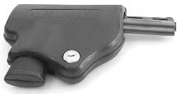	h. Gun cabinet	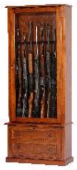
i. Small lock-box/hard case	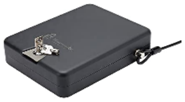	j. Other key/PIN/dial locking device:	[describe]
**Biometric locking device (e.g., fingerprint)**			
k. Cable lock	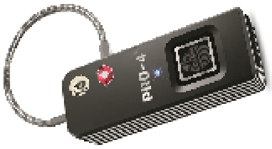	l. In-vehicle lock	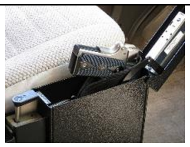
m. Trigger lock	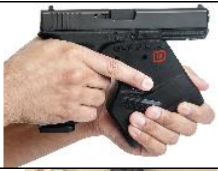	n. Gun safe	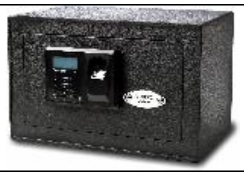
o. Clamshell/"life jacket"	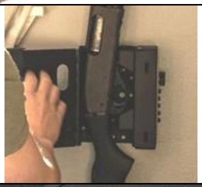	p. Gun cabinet	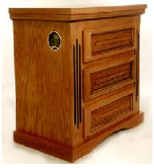
q. Small lock-box/hard case	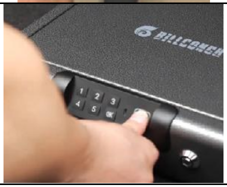	r. Other biometric locking device:	[describe]

© 2023 Anestis MD et al. *JAMA Network Open.*
