# Supplementary material for: Assessment of Firearm Storage Practices in the US, 2022
Source: JAMA Netw Open. 2023 Mar 2;6(3):e231447. doi: 10.1001/jamanetworkopen.2023.1447 (PMC9982690; doi:10.1001/jamanetworkopen.2023.1447)
Supplement: Supplement 2. — Data Sharing Statement [file jamanetwopen-e231447-s002.pdf]

Data Sharing Statement

Anestis. Assessment of Firearm Storage Practices in the US, 2022. *JAMA Netw Open*.
Published March 02, 2023. doi:10.1001/jamanetworkopen.2023.1447

Data
Data available: No
